# Expression Analysis of Chlorophyll-Degradation-Related Genes in *Prunus persica* L. Peel and the Functional Verification of Key Genes

**DOI:** 10.3390/plants14030312

**Published:** 2025-01-21

**Authors:** Xin Liu, Xiaoyu Zhang, Junren Meng, Ang Li, Wenyi Duan, Shihang Sun, Lei Pan, Wenfang Zeng, Zhiqiang Wang, Liang Niu

**Affiliations:** 1 Zhengzhou Fruit Research Institute, Chinese Academy of Agricultural Sciences/National Peach & Grape Improvement Center, Zhengzhou 450009, China; 1710141078@vip.henu.edu.cn (X.L.); 82101225105@caas.cn (X.Z.); 82101219216@caas.cn (J.M.); 1125351664la@gmail.com (A.L.); duanwenyi@caas.cn (W.D.); sunshihang@caas.cn (S.S.); panley@126.com (L.P.); zengwenfang@caas.cn (W.Z.); wangzhiqiang@caas.cn (Z.W.); 2 Zhongyuan Research Center, Chinese Academy of Agricultural Sciences, Xinxiang 453004, China

**Keywords:** chlorophyll degradation, fruit peel, *Prunus persica* L., *PpPAO* gene, *PpSGR* gene

## Abstract

With the evolution of consumer purchasing power and consumption concepts, external attributes such as fruit size, color, and peel smoothness have emerged as pivotal determinants influencing purchasing preferences; among these, the background color of the fruit peel exerts a considerable impact on fruit esthetics. The background color of fruit peel is predominantly influenced by the chlorophyll content. Consequently, examining the degradation patterns of chlorophyll in *Prunus persica* L. peel holds significant importance for cultivating varieties with a cleaner peel background color. In this study, *Prunus persica* L. CP14 and 20–29 were selected as experimental materials to evaluate the peel color variation and chlorophyll content during fruit development. Samples collected from three developmental stages of CP14 and 20–29 underwent transcriptome sequencing. Kyoto Encyclopedia of Genes and Genomes enrichment analysis identified chlorophyll-degradation-related genes within the purine metabolism pathway. Quantitative polymerase chain reaction analysis of chlorophyll degradation gene expression pinpointed *PpPAO* and *PpSGR* as likely key genes involved in chlorophyll degradation in *Prunus persica* L. Transient transformation assays in *Nicotiana benthamiana* leaves further substantiated that *PpPAO* and *PpSGR* markedly reduce chlorophyll levels. Yeast two-hybrid experiments also demonstrated an interaction between *PpPAO* and *PpSGR*.

## 1. Introduction

*Prunus persica* L., a stone fruit tree with significant economic importance, yields drupes that are highly valued globally by consumers due to their rich flavors and notable nutritional content, establishing it as one of the most widely consumed fruits in numerous countries. As consumer purchasing power and consumption preferences evolve, fruit quality has increasingly been regarded as a key determinant in shaping consumer behavior [1]. Peel coloration, being a vital aspect of fruit appearance quality, alongside color fading, has gained prominence as a central theme in related research.

In recent years, significant advancements have been made in elucidating the primary chlorophyll degradation pathway in plants. Within this process, chlorophyll b undergoes an initial conversion into 7-hydroxymethyl chlorophyll a, catalyzed by chlorophyll b reductase, which is encoded by the NON-YELLOW COLORING1 (NYC1) and NYC1-LIKE (NOL) genes [2]. Following this, 7-hydroxymethyl chlorophyll a is reduced to chlorophyll a via the activity of 7-hydroxymethyl chlorophyll a reductase [3]. Subsequently, chlorophyll a is transformed into pheophorbide a through two distinct routes: (1) through the combined action of chlorophyllase and magnesium-dechelatase (MDCase) [4] and (2) through catalysis by MDCase and pheophytinase [5]. In both pathways, pheophorbide a emerges as a shared intermediate, which is further degraded by pheophorbide a oxygenase (PAO) and red chlorophyll catabolite reductase (RCCR) [6]. PAO serves as a critical enzyme in the chlorophyll degradation pathway, and its catalysis of the oxidative ring-opening reaction of the porphyrin ring constitutes a pivotal step in chlorophyll breakdown. As a result, this degradation pathway is also referred to as the PAO pathway [7]. The identification of stay-green (SGR) and SGRL genes marked a substantial advancement in the understanding of chlorophyll degradation regulation [8]. These genes are implicated in the chlorophyll degradation pathway, facilitating the formation of complexes with chlorophyll degradation enzymes and the binding to photosystem II to form the SGR-CCE-LHC II complex, thereby enhancing chlorophyll degradation [9].

During fruit ripening, notable changes in coloration are widely regarded as key indicators of maturity and quality across various fruit species [10]. Chlorophyll catabolic enzymes (CCEs) and SGR proteins have been shown to serve prominent functions in this process [11]. The expression of PAO genes is markedly enhanced during senescence. For instance, BoPAO expression is upregulated during post-harvest senescence in broccoli [12], while AtPAO expression gradually increases in *Arabidopsis thaliana* throughout senescence, peaking during natural senescence [13]. The functions of SGR genes have been the focus of extensive research across diverse species. In *Litchi chinensis* L., the silencing of *LcSGR* markedly suppressed the expression of *LcNYC*, *LcPPH2*, and *LcPAO*, leading to delayed chlorophyll degradation in the peel of *L. chinensis* L. [14]. Investigations into *Lolium perenne* L. have revealed that *LpSGR* is localized to chloroplasts and interacts with other CCEs. Shimoda et al. demonstrated that SGR proteins exhibit MDCase activity, a function critical for chlorophyll degradation as it facilitates the removal of magnesium ions from chlorophyll molecules [15]. *Citrus sinensis* research reported that mutations of *CsSGR* result in the loss of chlorophyll degradation functionality [16]. Recent findings in *Glycine max* L. suggest that the absence of *GmSGR* may prevent seeds of *G max* L. from turning yellow, causing them to remain green [17]. These results indicate that overexpression of SGR may activate CCE genes, thereby regulating chlorophyll degradation in leaves. Furthermore, research on *Actinidia chinensis* has revealed that chlorophyll degradation-related genes, such as *SGR2*, are expressed at markedly higher levels in golden-flesh varieties compared with green varieties, resulting in earlier and more pronounced chlorophyll degradation [18]. Nonetheless, the mechanisms underlying chlorophyll degradation in many fruit species remain inadequately explored. Furthermore, within the green-fleshed kiwifruit, a structural variation is manifested in the promoter region of the *AcBCM* gene. This particular variation induces an elevated expression level of the *AcBCM* gene during the later developmental stage of the fruit, subsequently exerting an influence on the activity of *AcSGR2*, a pivotal enzyme in the chlorophyll catabolic process. Consequently, the green-fleshed varieties are still capable of maintaining a relatively high chlorophyll content subsequent to fruit ripening, thus exhibiting a unique green coloration [19]. Utilizing the citrus “Newhall” fruit as a model for the study of chlorophyll degradation, it was uncovered that the ethylene-responsive factor *CitERF13* directly binds to the citpph promoter and potentiates its activity. Its transient and stable overexpression in both tobacco leaves and citrus peel culminate in rapid chlorophyll degradation [20]. In apples, the ethylene-responsive transcription factor *MdERF17* is proficient at binding to chlorophyll degradation genes, thereby regulating the chlorophyll degradation process in the peel [21]. The Cavendish banana exhibits green-ripening at temperatures exceeding 24 °C, whereas the Bluggoe fruit turns completely yellow. The expression of stay-green 1 (*SGR1*), a key gene involved in chlorophyll degradation, is diminished in the Cavendish banana but is induced in the Bluggoe fruit, which correlates with the fruit degreening patterns [22].

RNA sequencing (RNA-seq) is a technique that employs high-throughput sequencing technologies to investigate transcript expression profiles in specific cells or tissues under defined temporal or conditional settings. The Illumina sequencing platform, which is among the most widely utilized high-throughput sequencing methods, facilitates the rapid and precise sequencing of numerous transcripts, thereby driving substantial progress in the study of gene function and structure. Transcriptome analysis has been successfully employed in *C. sinensis* [23], *Solanum lycopersicum* [24], and *Brassica rapa* L. [25]. Utilizing transcriptome and chlorophyll content analyses in *Zoysia japonica* Steud., it was determined that ethephon pretreatment enhanced leaf chlorophyll levels, while the suppression of chlorophyll degradation induced by cold stress was achieved through the downregulation of *ZjPAO*, *ZjRCCR*, and *ZjSGR* expression [26].

The peel color of *P. persica* L. fruit represents one of the key factors influencing its edible quality and market value. Although chlorophyll degradation and its associated gene regulation have been explored in certain plant species [27], comprehensive studies detailing the patterns of chlorophyll degradation and the expression of related genes in *P. persica* L. remain scarce. In this study, transcriptome analysis was employed to identify critical genes implicated in chlorophyll degradation in *P. persica* L. Quantitative real-time polymerase chain reaction (qRT-PCR) was conducted to thoroughly analyze the expression patterns of chlorophyll degradation genes in the peel of Zhongtao 14 and 20–29. The relationship between the expression of these genes, chlorophyll content, and fruit color variations was examined. Through transient expression assays of the key genes *PpPAO* and *PpSGR* in *Nicotiana benthamiana*, it was demonstrated that *PpPAO* and *PpSGR* were capable of inducing chlorophyll degradation in *N. benthamiana* leaves. Additionally, yeast two-hybrid (Y2H) experiments confirmed the interaction between the chlorophyll degradation genes *PpPAO* and *PpSGR* in *P. persica* L.

## 2. Results

### 2.1. Analysis of Color Differences and Chlorophyll Changes in P. persica L. Peel Before Ripening

**Figure 1:**
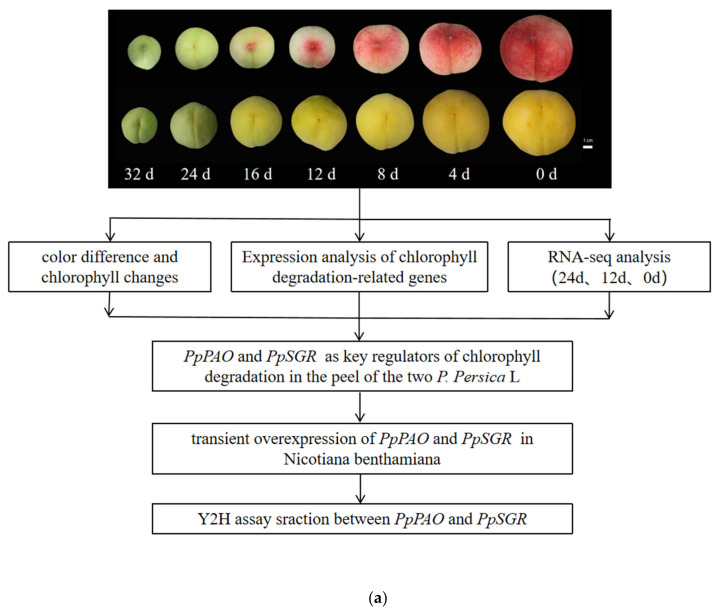

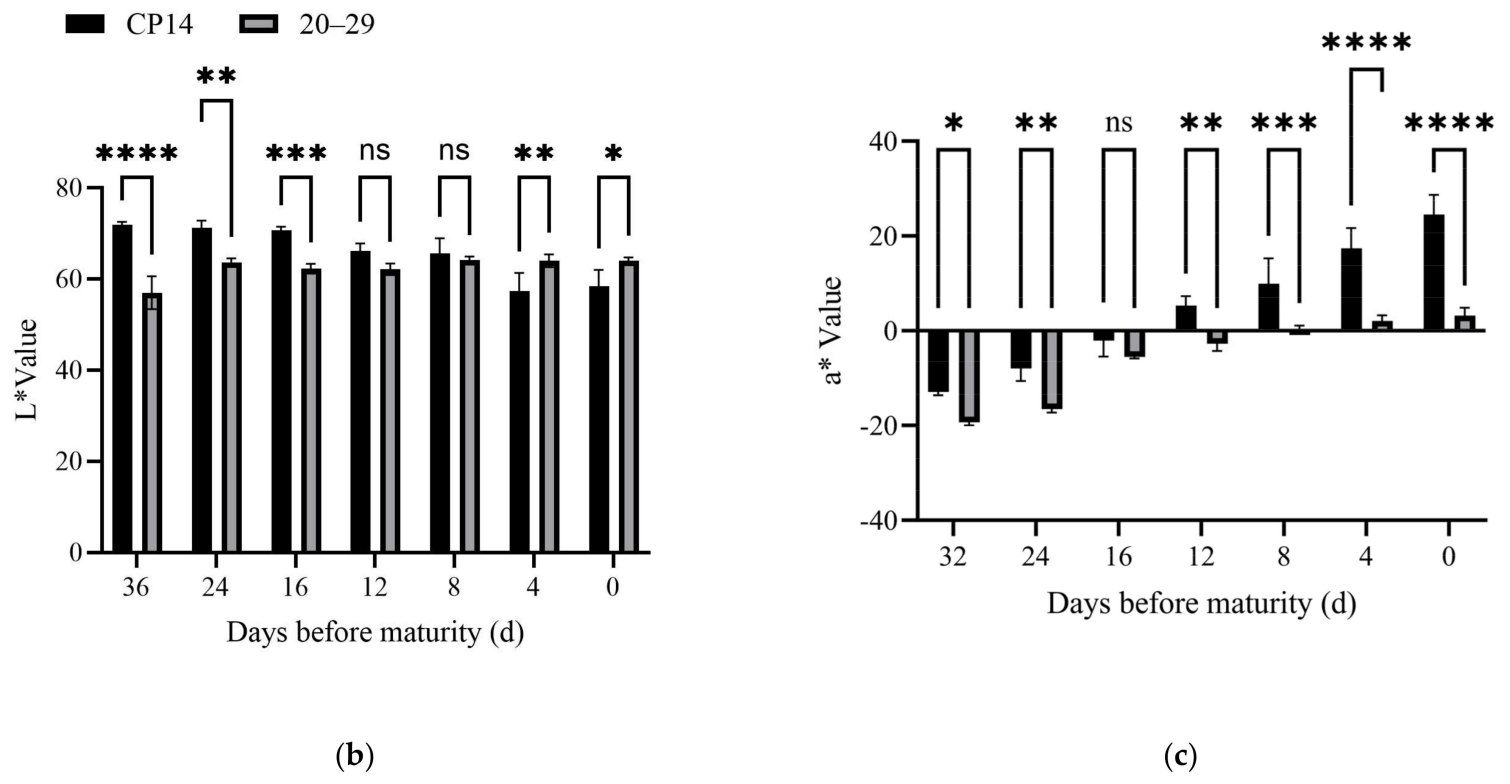

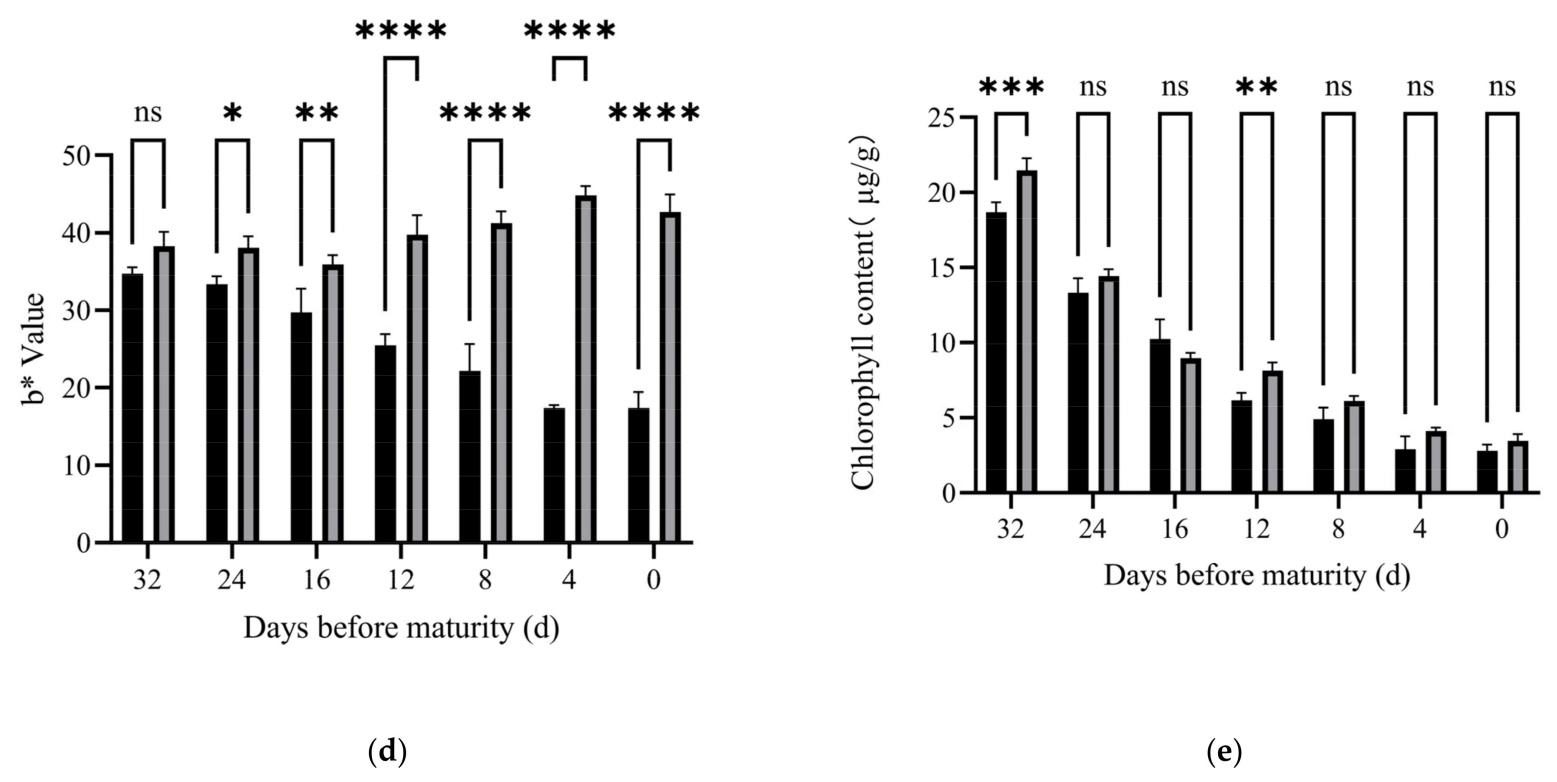
(**a**) Phenotypes of fruits at 32-0 DBM (bar = 1 cm) and the experimental workflow in this study; (**b**) Changes in peel color difference L* values of CP14 and 20–29 before fruit ripening; (**c**) Changes in peel color difference a* values of CP14 and 20–29 before fruit ripening; (**d**) Changes in peel color difference b* values of CP14 and 20–29 before fruit ripening; (**e**) Changes in chlorophyll content of CP14 and 20–29 before fruit ripening. Bars represent ± SD (*n* = 3). Asterisks indicate a significant difference between CP14 and 20–29 (* *p* < 0.05, ** *p* < 0.01, *** *p* < 0.001, and **** *p* < 0.0001 level); “ns” means not significant.

The alterations in the color phenotype observed in Zhongtao 14 and 20–29 prior to maturation are presented in Figure 1a. The peel of Zhongtao 14 underwent a gradual transition from green to red 12 days before maturity (DBM), followed by a pronounced intensification of its coloration. In contrast, the peel of lines 20–29 began exhibiting a light yellow hue at 16 and 12 DBM.

Variations in the color differences of *P. persica* L. peel were examined over the 32 days and within the month preceding fruit maturation. The L* value (Figure 1b) of the Zhongtao 14 peel progressively declined between 32 and 16 DBM, followed by a more pronounced reduction at 12 DBM, coinciding with the initiation of peel coloration. In contrast, the L* value of lines 20–29 increased due to the lack of coloration and subsequently stabilized. The changes in the a* values (Figure 1c) for both Zhongtao 14 and 20–29 exhibited S-shaped growth patterns during the month prior to fruit maturity. For Zhongtao 14, beginning at 16 DBM, the green coloration of the fruit gradually diminished while the red coloration emerged, leading to a steady increase in the a* value. During the 16–12 DBM period, the a* values of both genotypes either transitioned from negative to positive or approached zero, signifying a pivotal phase of color transformation. The b* value (Figure 1d) of Zhongtao 14 displayed an overall decreasing trend during the 32 days leading up to fruit maturity. In contrast, the b* value of lines 20–29 showed a continuous increase as the fruit matured, with a slight decline observed at full maturity. The measured color difference values aligned with the phenotypic color changes observed in both Zhongtao 14 and 20–29.

The chlorophyll content in the peel of *P. persica* L. for both Zhongtao 14 and 20–29 was found to follow a declining trend during fruit development and ripening, as illustrated in Figure 1e. A marked reduction in chlorophyll content was recorded during the period from 32 to 16 DBM. Between 12 and 8 DBM, the chlorophyll content in all varieties decreased to levels below 10 μg/g, accompanied by a visible reduction in the green coloration in both varieties/lines. From 8 DBM to full maturity, the rate of chlorophyll degradation slowed in both varieties. At fruit maturity, Zhongtao 14 displayed a lower chlorophyll content in comparison with lines 20–29.

### 2.2. Mining Key Genes Involved in Chlorophyll Degradation in P. persica L.

**Figure 2:**
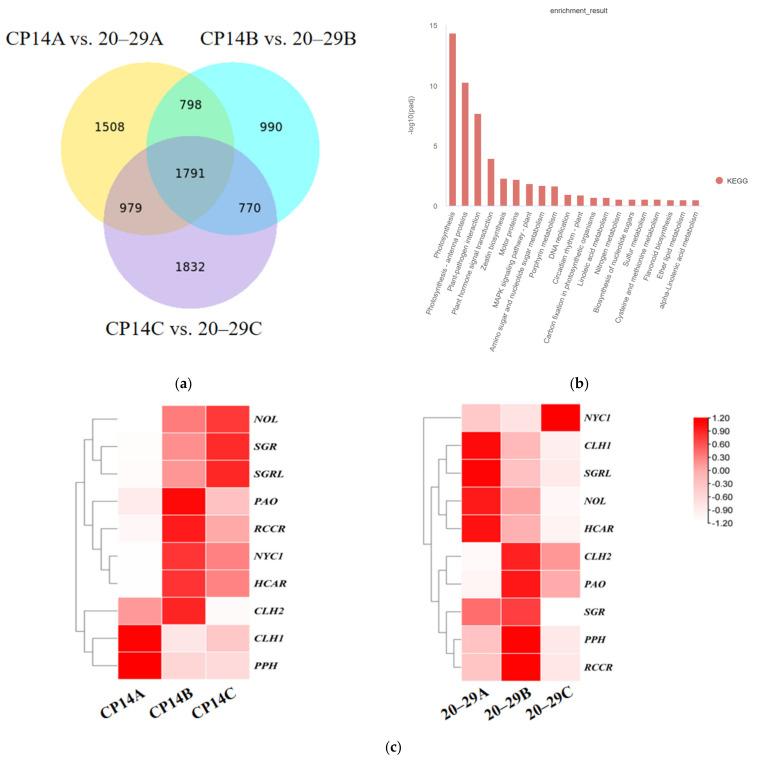
(**a**) Distribution of differential genes shown as a Venn diagram (CP14 vs. 20–29); (**b**) Enriched KEGG terms of CP14 vs. 20–29; (**c**) DEGs involved in chlorophyll degradation identified in the RNA-seq data of *P. persica* L. pericarp.

RNA-seq analysis was conducted on samples from three developmental stages (24, 12, and 0 days before maturity) of CP14 and 20–29. The RNA-seq data identified 1791 differentially expressed genes (DEGs) shared across all three stages between CP14 and 20–29 (Figure 2a), which were then subjected to the Kyoto Encyclopedia of Genes and Genomes (KEGG) pathway enrichment analysis. In the KEGG enrichment of downregulated genes (Figure 2b), chlorophyll degradation genes were enriched in the porphyrin metabolism pathway. Subsequently, attention was directed to the identified DEGs associated with the chlorophyll degradation pathway (Figure 2c). The expression levels of *PpPAO* and *PpSGR* genes were observed to be relatively high in both varieties, resulting in their selection as key genes for further investigation.

### 2.3. Expression Analysis of Chlorophyll-Degradation-Related Genes During Late Fruit Development

**Figure 3:**
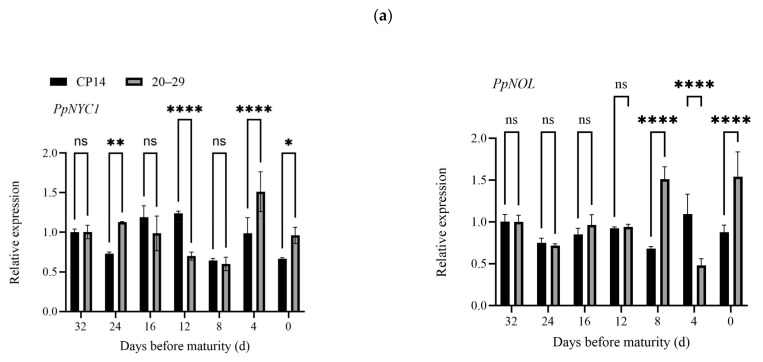

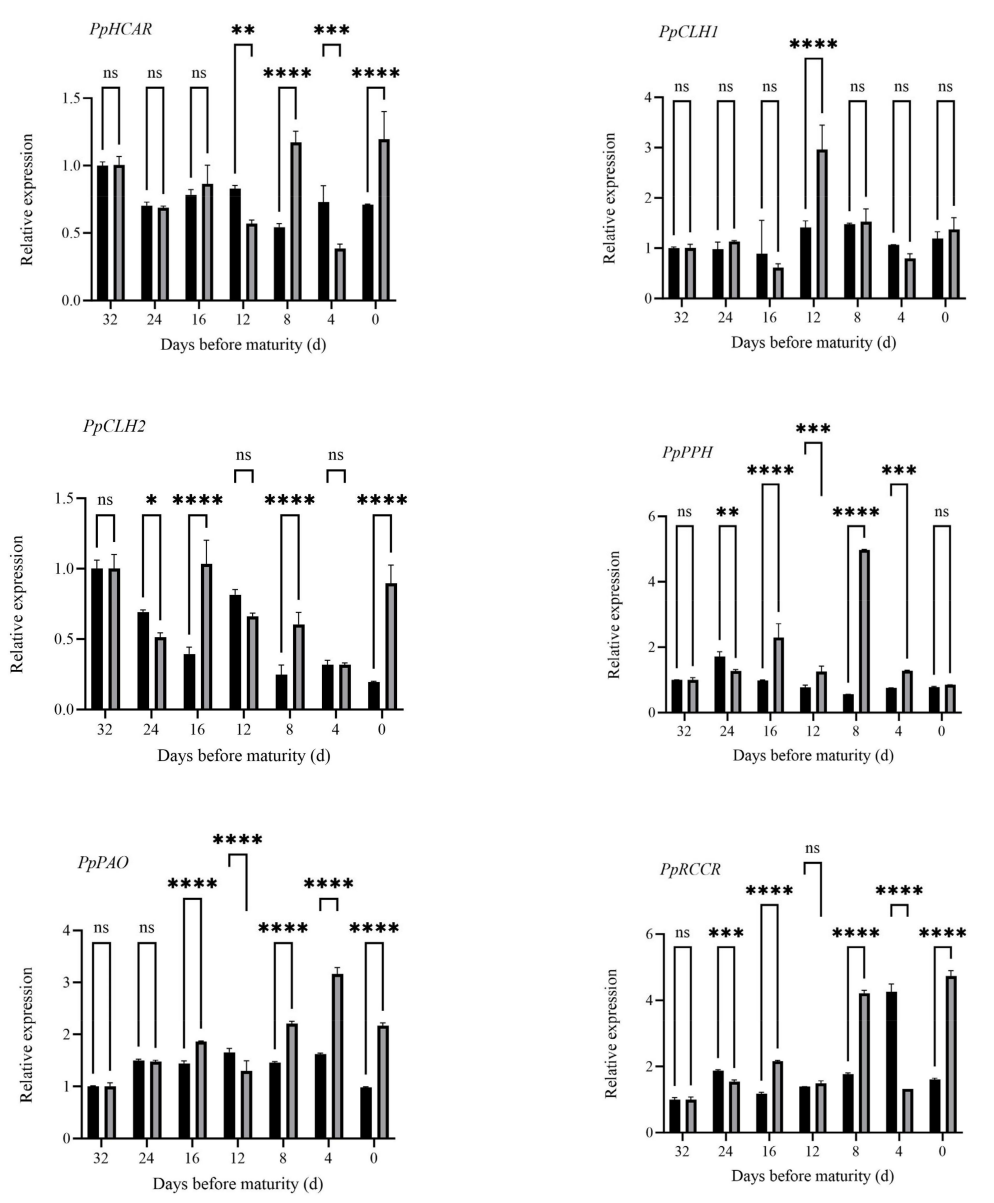

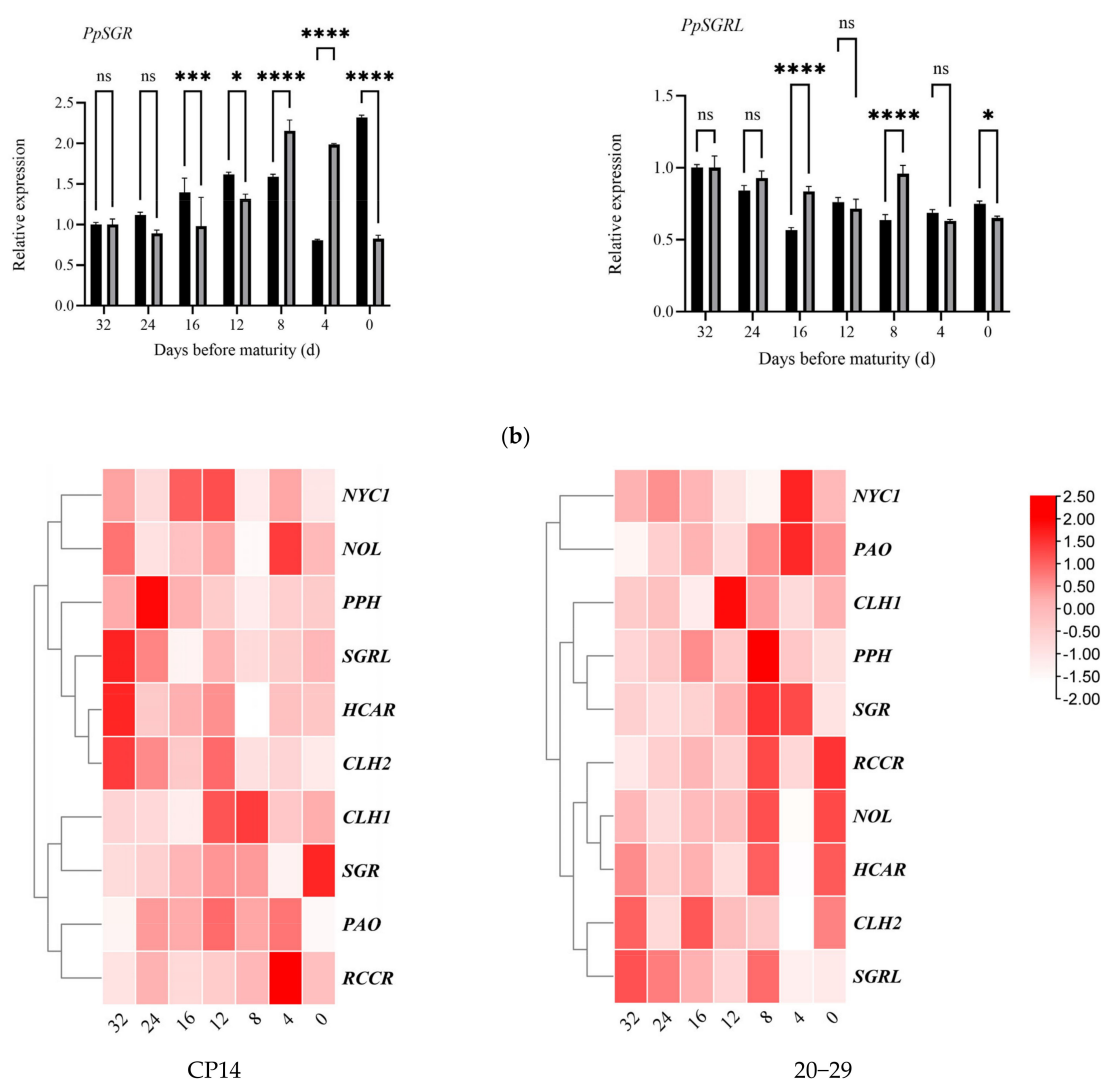
(**a**) Expression of chlorophyll degradation genes; (**b**) Cluster analysis of chlorophyll degradation genes in different *P. persica* L. varieties. Bars represent ± SD (*n* = 3). Asterisks indicate a significant difference between CP14 and 20–29 (* *p* < 0.05, ** *p* < 0.01, *** *p* < 0.001, and **** *p* < 0.0001 level); ”ns” means not significant.

The relative expression levels of 10 genes (*PpNYC1*, *PpNOL*, *PpHCAR*, *PpCLH1*, *PpCLH2*, *PpPPH*, *PpPAO*, *PpRCCR*, *PpSGR*, and *PpSGRL*) associated with chlorophyll degradation in two *P. persica* L. varieties/lines were quantified using qRT-PCR. As illustrated in Figure 3a, the expression levels of *PpNYC1*, *PpNOL*, and *PpHCAR* decreased during maturation in Zhongtao 14 but increased in 20–29 during the same period. *PpCLH1* exhibited elevated expression at the color-changing stage (12 days before maturity) in both Zhongtao 14 and 20–29. In 20–29, *PpPPH* and *PpRCCR* displayed significant increases at 8 DBM, whereas *PpRCCR* showed a marked increase at 4 DBM in Zhongtao 14. The expression levels of *PpCLH2* and *PpSGRL* progressively declined throughout fruit development in both varieties/lines. As the peel’s chlorophyll degraded and the fruit reached full maturity, the expression levels of *PpPAO* and *PpSGR* exhibited a substantial increase. These findings led to the hypothesis that *PpPAO* and *PpSGR* might serve as the primary genes responsible for chlorophyll degradation before fruit maturity in these two *P. persica* L. varieties/lines.

The results of the cluster analysis (Figure 3b) demonstrated that *PpSGR* was clustered with *PpCLH1*, *PpPAO*, and *PpRCCR* in CP14, whereas in line 20–29, *PpSGR* was grouped with *PpCLH1* and *PpPPH*. The correlation analysis between chlorophyll content and its associated degradation genes (Table 1) revealed a significant positive correlation between *PpCLH2* expression and chlorophyll content in line 20–29, while *PpPAO* expression displayed a significant negative correlation with chlorophyll content in CP14.

In summary, the qRT-PCR results demonstrated that the expression levels of *PpPAO* and *PpSGR* were markedly upregulated during 8–0 DBM. The cluster analysis indicated that *PpSGR* was clustered with *PpCLH1*, *PpPAO*, and *PpRCCR* in CP14. The correlation analysis revealed a significant negative correlation between *PpPAO* expression and chlorophyll content in CP14. Consequently, within the month preceding fruit maturation, chlorophyll degradation in the peel of *P. persica* L. was associated with *PpPAO* and *PpSGR*, with variations identified among different *P. persica* L. varieties. These findings preliminarily suggest that *PpPAO* and *PpSGR* serve as key regulators of chlorophyll degradation in the peel of the two *P. persica* L. varieties/lines prior to fruit maturation.

### 2.4. Phenotypic Analysis following Transient Overexpression of PpPAO and PpSGR in N. benthamiana

**Figure 4:**
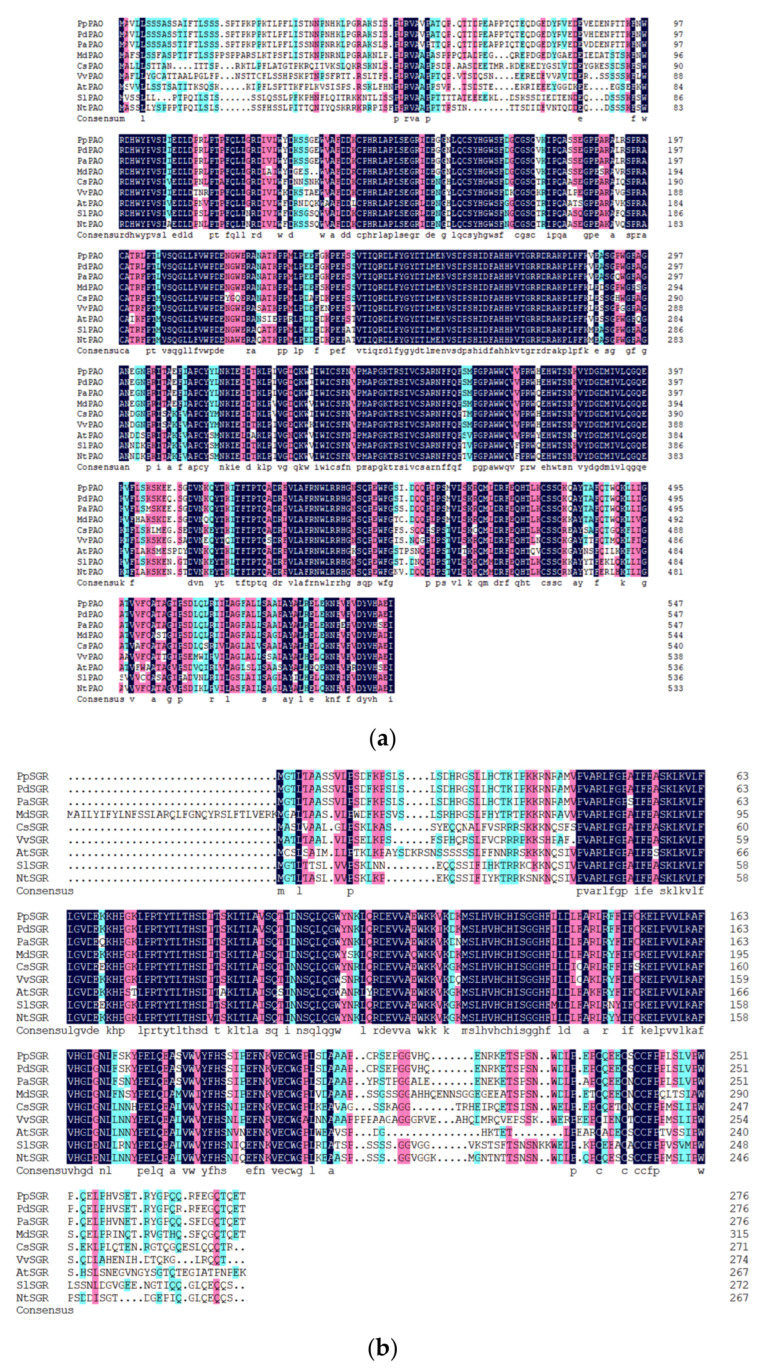
(**a**) Alignment of the amino acid sequences of PAO proteins from eight species. (**b**) Alignment of the amino acid sequences of SGR proteins from eight species. *At*, *Arabidopsis thaliana*; *Nt*, *Nicotiana tabacum*; *Sl*, *Solanum lycopersicum*; *Vv*, *Vitis vinifera*; *Cs*, *Citrus sinensis*; *Md*, *Malus domestica*; *Pa*, *Prunus avium*; *Pd*, *Prunus dulcis*; *Pp*, *Prunus persica*.

The amino acid sequence alignment of PAO from various species (Figure 4a) revealed that the *PpPAO* protein from *P. persica* L. exhibits a high degree of homology with PAO proteins from other species. DNAMAN analysis indicated that the sequence identity among *PpPAO* from *P. persica* L., *PdPAO* from *Prunus armeniaca* L., *PaPAO* from *Prunus pseudocerasus*, *MdPAO* from *Malus pumila* Mill., *CsPAO* from *Citrus reticulata* Blanco, *VvPAO* from *Vitis vinifera* L., *AtPAO* from *A. thaliana*, *SlPAO* from *S. lycopersicum* L., and *NtPAO* from *N. benthamiana* was 83.81%. Among these, *PpPAO* exhibited the highest homology (99.3%) with *PdPAO* from *Prunus armeniaca*, supporting their close phylogenetic relationship. The notable similarity (98.5%) between *PaPAO* from *P. pseudocerasus* and *PpPAO* highlights the strong conservation of PAO among higher plants.

The amino acid sequence alignment of SGR from different species (Figure 4b) demonstrated that the *PpSGR* protein from *P. persica* L. shares substantial homology with SGR proteins from other species. DNAMAN analysis revealed that the sequence identity among *PpSGR* from *P. persica* L., *PdSGR* from *P. armeniaca* L., *PaSGR* from *P. pseudocerasus*, *MdSGR* from *M. pumila* Mill., *CsSGR* from *C. reticulata* Blanco, *VvSGR* from *V vinifera* L., *AtSGR* from *A. thaliana*, *SlSGR* from *S. lycopersicum* L., and *NtSGR* from *N. benthamiana* was 68.48%. Notably, *PpSGR* exhibited the highest homology (99.3%) with *PdSGR* from *P. armeniaca* L., further confirming the close phylogenetic relationship between *P. persica* L. and *P. armeniaca* L. The high similarity (96.4%) between *PaSGR* from *P. pseudocerasus* and *PpSGR* underscores the strong conservation of SGR within the *Rosaceae* family.

**Figure 5:**
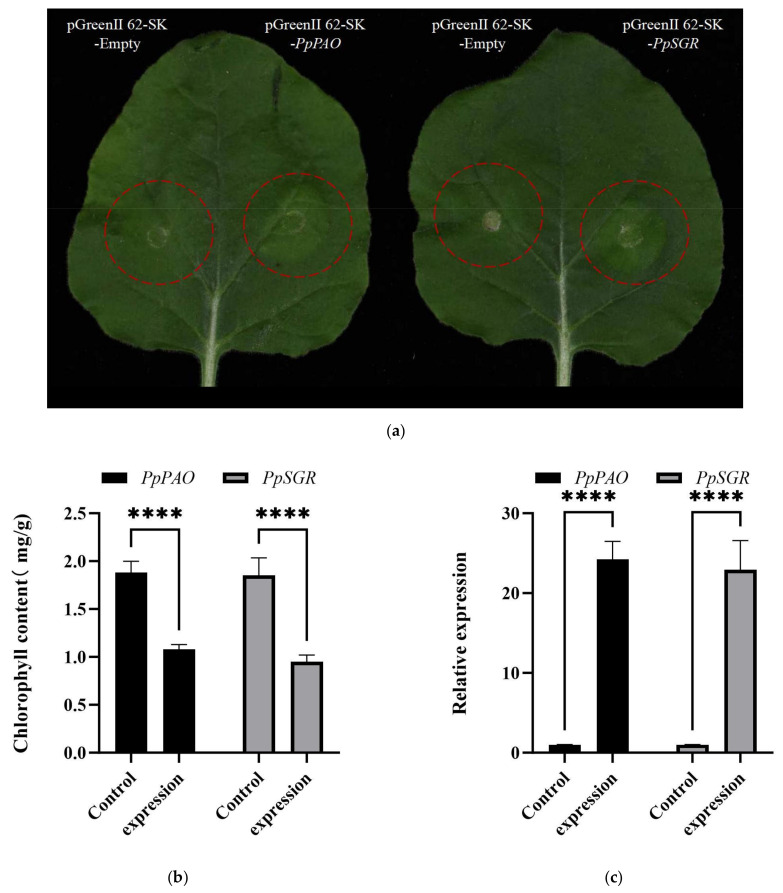
(**a**) The phenotype of *PpPAO* and *PpSGR* genes in the instant transformation experiment in *N. benthamiana* (**b**). The chlorophyll content of *PpPAO* and *PpSGR* genes in the instant transformation experiment in *N. benthamiana*. (**c**). The expression levels of *PpPAO* and *PpSGR* genes in the transient transformation experiment in *N. benthamiana*. Bars represent ± SD (*n* = 3). Asterisks indicate a significant difference in expression compared with the control (**** *p* < 0.0001 level).

Under identical growth conditions, with the pGreenII 62-SK empty vector serving as a control, significant chlorophyll degradation was observed in *N. benthamiana* leaves infiltrated with *PpPAO* and *PpSGR* compared with the WT (Figure 5a).

To examine the impact of transient injection of *PpPAO* and *PpSGR* genes on the chlorophyll content in *N. benthamiana*, the chlorophyll content of leaves was measured (Figure 5b). The findings demonstrated that the chlorophyll content in WT *N. benthamiana* leaves was markedly higher than that in transiently injected leaves. The expression of *PpPAO* and *PpSGR* was detected in the transiently injected *N. benthamiana* leaves through qRT-PCR analysis. Compared with the WT control, the expression levels of *PpPAO* and *PpSGR* in the injected leaves were upregulated by 24-fold and 22-fold, respectively (Figure 5c).

### 2.5. Y2H Analysis of P. persica L. PAO and SGR

**Figure 6:**
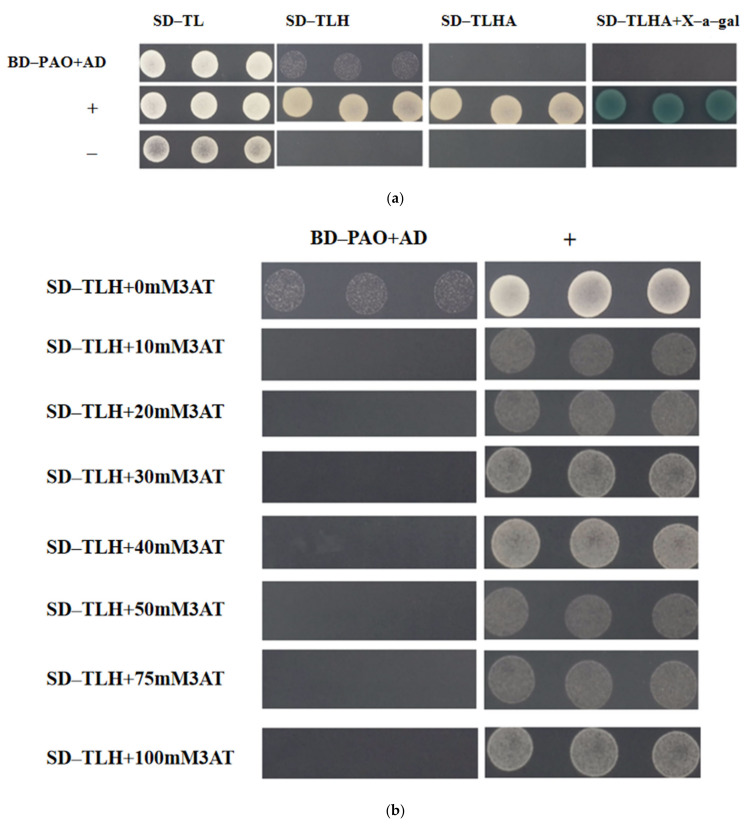

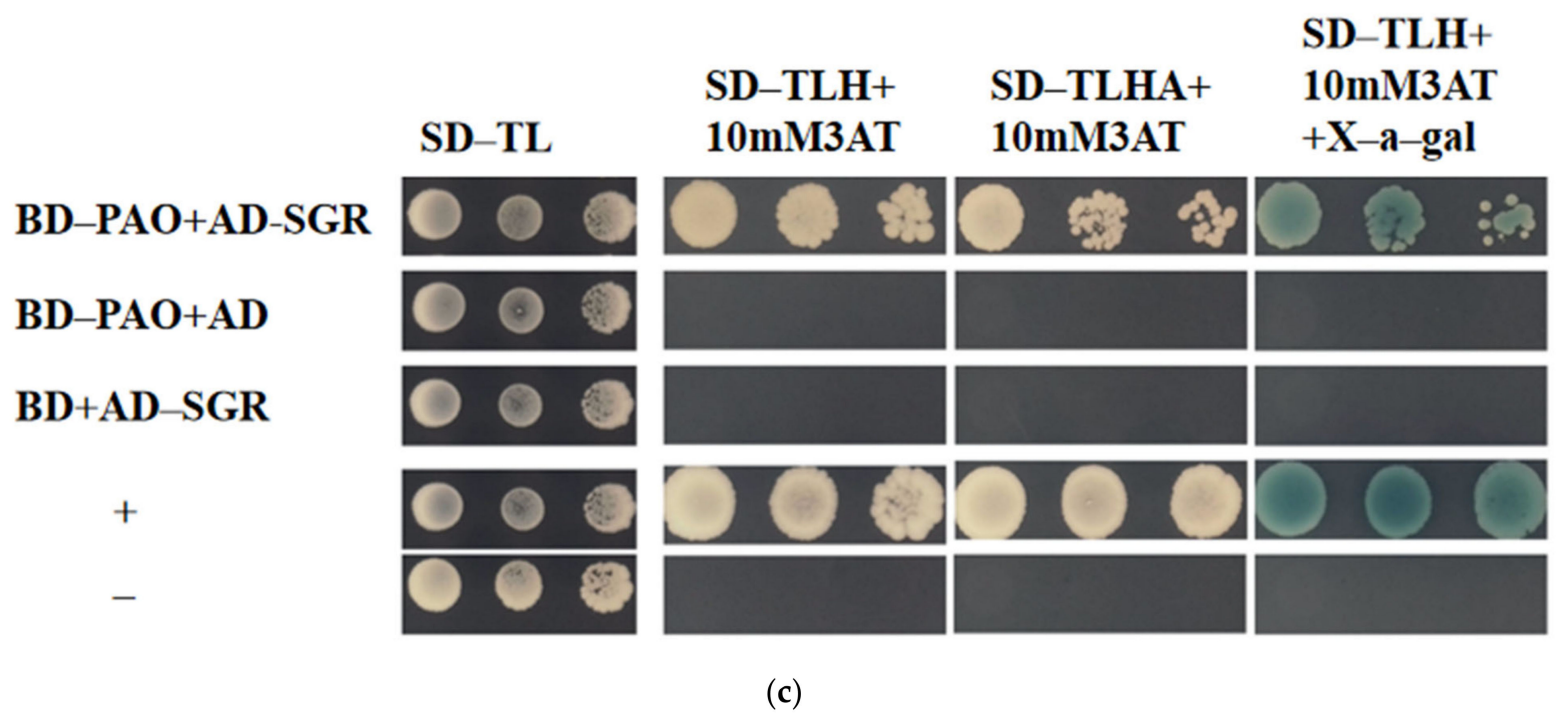
(**a**) PAO self-activation detection. (**b**) Results of PAO self-activation inhibition at the 3AT inhibitory concentration of 10 mM. (**c**) Y2H assay interaction between PAO and SGR.

As illustrated in Figure 6a, the control strain with the negative control exhibited growth on SD/-Trp/-Leu plates but failed to grow normally on SD-TLH plates. The experimental group displayed normal growth on SD/-Trp/-Leu/-His plates but was unable to grow on SD-TLH plates, indicating that pGBKT7-PAO demonstrated self-activation.

The clones of pGBKT7-PAO + pGADT7 and pGADT7-largeT + pGBKT7-p53 were resuspended in 2 mL of sterile ddH_2_O and spotted onto nutrient-deficient plates, including SD-TLH, SD-TLH + 10 mM 3AT, SD-TLH + 20 mM 3AT, SD-TLH + 30 mM 3AT, SD-TLH + 40 mM 3AT, SD-TLH + 50 mM 3AT, SD-TLH + 75 mM 3AT, and SD-TLH + 100 mM 3AT. As depicted in Figure 6b, no self-activation was detected for pGBKT7-PAO at the 3AT inhibitory concentration of 10 mM.

The experimental group containing the pGBKT7-PAO and pGADT7-SGR strains exhibited normal growth on SD-TL, SD-TLH + 10 mM 3AT, SD-TLHA + 10 mM 3AT, and SD-TLHA + 10 mM 3AT + X-α-gal deficient plates and displayed blue coloration on SD-TLHA + 10 mM 3AT + X-α-gal medium. In contrast, the control groups, consisting of pGBKT7-PAO and pGADT7, pGBKT7 and pGADT7-SGR, and the negative control pGADT7-largeT + pGBKT7-laminC strains, exhibited normal growth only on SD-TL plates but failed to grow on SD-TLH supplemented with 10 mM 3AT, SD-TLHA with 10 mM 3AT, and SD-TLHA with 10 mM 3AT and X-α-gal deficient plates (Figure 6c). These results suggest an interaction between pGBKT7-PAO and pGADT7-SGR.

## 3. Discussion

### 3.1. Expression of Chlorophyll Degradation Genes in P. persica L. Peel

In recent years, research on chlorophyll degradation in the peel of *P. persica* L. has primarily concentrated on physiological aspects. Although transcriptomic studies specifically addressing chlorophyll degradation in *P. persica* L. fruit remain limited, related investigations have been carried out in other species. Lai et al. conducted transcriptome sequencing on *L. chinensis* L. peel at three distinct developmental stages and identified genes encoding enzymes associated with chlorophyll degradation and flavonoid biosynthesis [28], thereby providing new insights into the molecular mechanisms underlying chlorophyll degradation. Through transcriptomic and genomic analyses of *Apium graveolens* L., Song et al. identified 16 key genes involved in chlorophyll regulation [29]. Wang et al. performed transcriptome sequencing on dark-green and light-green *Cucumis sativus* peels, identifying 4516 DEGs between the two samples [30]. Further analysis revealed that the reduced expression of chlorophyll biosynthesis genes (such as *chlM* and *POR*) contributed to a decrease in chlorophyll content in the peel. In this study, mature fruit peels from Zhongtao 14 and lines 20–29 were utilized as experimental materials. Through transcriptome sequencing, functional enrichment analysis, and screening of chlorophyll-degradation-related genes, transcriptomic data were provided to support future studies on chlorophyll degradation in *P. persica* L. fruit.

In higher plants, alterations in the expression levels and enzyme activities of PAO genes have been linked to chlorophyll degradation [31]. In *L. chinensis* L., the expression levels of *LcPAO* and *LcSGR* progressively increase during fruit development, with markedly lower expression levels observed in ”Feizixiao” compared with ”Nuomici” and the latter exhibiting complete chlorophyll degradation [14]. In this study, *PpPPH* and *PpPAO* exhibited significant increases at 8 DBM and 4 DBM, respectively, in lines 20–29, while *PpPPH* also showed a notable increase in CP14. The cluster analysis results (Figure 3b) demonstrated that *PpSGR* clustered with *PpCLH1*, *PpPAO*, and *PpRCCR* in CP14, whereas in line 20–29, *PpSGR* was clustered with *PpCLH1* and *PpPPH*. The correlation analysis between chlorophyll content and its degradation genes (Table 1) revealed a significant positive correlation between *PpCLH2* expression and chlorophyll content in line 20–29, while *PpPAO* exhibited a significant negative correlation with chlorophyll content in CP14. SGR is generally involved in chlorophyll degradation during plant leaf senescence and serves a vital function in the development of various plant organs.Taking rice as the research object, it was found that the expression of the *SGR* gene in rice was specifically upregulated under magnesium-deficient conditions. *SGR*-mediated chlorophyll degradation not only promoted the transfer of magnesium from mid-stage leaves to young leaves but also played a role in photo-oxidative protection under magnesium-deficient conditions [32]. Sakuraba et al. indicated that during *A. thaliana* leaf senescence, *SGR1* positively mediates chlorophyll degradation, while overexpression of *SGRL* in *A. thaliana* results in early leaf yellowing [33]. Luo et al. found that studies have identified *SlSGR* as a key gene involved in chlorophyll degradation in *S. lycopersicum* L. [34]. Compared with WT fruits, *SlSGR* knockout lines in *S. lycopersicum* L. displayed markedly higher chlorophyll levels and a notably turbid brown coloration [35], highlighting *SlSGR*’s role in chlorophyll degradation. Chlorophyll degradation, which leads to peel degreening, is one of the most evident indicators of *P. persica* L. fruit ripening. The findings revealed that *PpPAO* and *PpSGR* exhibited higher expression levels during fruit ripening, with the chlorophyll content in lines 20–29 showing a negative correlation with *PpPAO*. This suggests that *PpPAO* and *PpSGR* may serve as key regulatory genes for chlorophyll degradation during *P. persica* L. fruit ripening. Therefore, further investigation into the regulatory roles of chlorophyll degradation genes before fruit ripening in *P. persica* L. is of considerable significance.

### 3.2. Function of Chlorophyll Degradation Genes

The construction of plant expression vectors and transient gene expression systems has emerged as a rapid and efficient approach for investigating gene functions, with widespread application across various plant species. Subcellular localization studies in *Ananas comosus* var. *bracteatus* L. have revealed that PAO operates within chloroplasts, and further experiments demonstrated that the overexpression of the PAO gene induces chlorophyll degradation in leaves [36]. The oxidation of pheophorbide a, a critical step in chlorophyll degradation, is catalyzed by PAO, an iron-containing and ferredoxin-dependent monooxygenase. Research has shown that inhibiting PAO activity prevents chlorophyll degradation [37]. Transcriptome and RT-qPCR analyses have indicated that the expression of *A. chinensis* SGRs is strongly influenced by light and developmental stages, which are regulated by plant growth regulators. This finding is supported by the identification of plant hormone-responsive cis-regulatory elements within the promoter region. In *AcSGR2* overexpression lines, reductions in Fv/Fm, SPAD values, and chlorophyll content were observed [38]. Overexpression of SGR in *Oryza sativa* L. reduced the number of thylakoid layers in chloroplasts and decreased the chlorophyll content in normally growing leaves, suggesting that SGR upregulation enhances chlorophyll degradation during leaf senescence [39].The *SlSGR1* gene in tomatoes was knocked out using CRISPR/Cas9 technology. It was found that the fruits after knockout showed a muddy brown color, and the contents of chlorophyll and carotenoids were significantly higher than those of wild-type fruits. Through in-depth transcriptome sequencing analysis, a total of 354 differentially expressed genes were identified between the wild-type and *SlSGR1* knockout lines. These results indicate that *SlSGR1* is involved in the color change of mature fruits through chlorophyll degradation and carotenoid biosynthesis [40]. RNA interference of *LpSGR* in *L. perenne* L. was found to block chlorophyll degradation, resulting in increased chlorophyll content and enhanced photochemical efficiency in senescing leaves [41]. Further studies in *L. perenne* L. demonstrated that the suppression or knockout of SGR expression delayed the degradation of chlorophyll a, yielding distinct SGR phenotypes during natural development or dark-induced senescence. Additionally, SGR-mediated chlorophyll metabolism was found to serve a crucial function in maintaining photosystem stability under heat stress [42]. These findings underscore the impact of SGR overexpression on plant chlorophyll content and photosynthesis.

In the present study, plant overexpression vectors were constructed to investigate the roles of *PpPAO* and *PpSGR* in chlorophyll degradation. Experimental results demonstrated that the overexpression of *PpPAO* and *PpSGR* led to chlorophyll degradation in *N. benthamiana* leaves. qRT-PCR analysis revealed that the expression levels of *PpPAO* and *PpSGR* were markedly upregulated in transiently injected *N. benthamiana* leaves compared with the control. Bioinformatics analysis of *PpPAO* and *PpSGR* revealed high sequence conservation among higher plants, emphasizing the conserved nature of chlorophyll degradation genes. Phylogenetic analysis indicated a close relationship between *P. armeniaca* L. and *P. pseudocerasus* with *P. persica* L. Alfalfa (*Medicago sativa*) has been established as a model leguminous plant. Its closely related species, also named Medicago sativa, represents the most widely cultivated forage legume crop across the United States. Relying on the *MtSGR* sequence, the SGR gene of alfalfa, designated as *MsSGR*, was successfully cloned, and subsequently, transgenic alfalfa lines were produced via RNA interference. The silencing of *MsSGR* resulted in the generation of transgenic alfalfa with the stay-green characteristic. This advantageous trait offers a promising opportunity for breeding high-quality alfalfa varieties that possess a more vibrant green appearance [43]. The functions of chlorophyll degradation genes unearthed in this research have furnished novel perspectives for cultivating peach varieties with optimal appearance attributes. For instance, during the development of certain commercial early-ripening peach cultivars, breeders anticipate that the fruits can swiftly degrade chlorophyll within a brief timeframe, thereby presenting an alluring hue and hitting the market prematurely. Future investigations could further probe the interaction webs among these CCGs (chlorophyll degradation genes) and other genes associated with fruit quality traits, precisely identifying each pivotal node within the intricate genetic regulatory network governing chlorophyll degradation with the aim of providing valuable benchmarks for more refined and efficacious fruit breeding.

The interaction between PAO and SGR has been previously demonstrated through Y2H experiments in *A. thaliana* and *L. chinensis* L. [9,14]. In this study, the interaction between *PpPAO* and *PpSGR* was also confirmed. These results provide a valuable foundation for further exploration of the molecular regulatory mechanisms and gene networks involved in chlorophyll degradation in the peel of *P. persica* L.

## 4. Methods

### 4.1. Plant Materials

The experimental materials were derived from the *P. persica* L. varieties/lines Zhongtao 14 and 20–29 maintained in the peach germplasm repository of the Zhengzhou Fruit Research Institute, Chinese Academy of Agricultural Sciences. The trees, planted in 2017 with a spacing of 1.0 m × 4.0 m, were cultivated under conventional management practices. From May to June 2023, beginning at 30 DBM, 30 fruits of uniform size, free from pests and diseases, and at a similar maturity stage were harvested from the middle–upper outer canopy of the trees. The fruits were photographed in the laboratory using a Nikon D700 camera (Nikon, Tokyo, Japan) under natural light against a black flocked fabric background. The samples were then grouped into sets of 10 fruits, with three replicates per set. Sampling was initially conducted at 8-day intervals, followed by 4-day intervals for subsequent collections. The fruit peel was carefully removed, rapidly frozen in liquid nitrogen, and stored at −80 °C for further analysis.

### 4.2. Chlorophyll Measurement

The chlorophyll content was ascertained utilizing UV spectrophotometry [44].

### 4.3. RNA Extraction, Library Construction, and RNA Sequencing

The transcriptome library was constructed by pooling equal amounts of RNA extracted from the fruit peel at each of the three developmental stages, while the three DEG libraries were prepared using individual RNA extracts from the fruit peel at the respective stages. Each DEG library contained equal quantities of RNA derived from three biological replicates at each stage. Total RNA from *P. persica* L. was isolated per the supplier’s instructions using the Plant Total RNA Extraction Kit for Polysaccharides and Polyphenols (DP441, Tiangen Biotech Co., Ltd., Beijing, China). RNA quality and purity were evaluated by 1% agarose gel electrophoresis, and the RNA concentration was determined using 1 μL of RNA in a NanoDrop2000 spectrophotometer (Thermo Scientific, Waltham, MA, USA). Sequencing and subsequent analyses were carried out on the Illumina platform by Novogene (Tianjin, China).

### 4.4. Sequence Alignment to the Reference Genome

The reference genome and gene model annotation files were retrieved directly from the genome website. The reference genome index was constructed using HISAT2 v2.0.5, and paired-end clean reads were aligned to the reference genome with the same version of HISAT2. HISAT2 was chosen as the mapping tool due to its ability to generate splicing junction databases from the gene model annotation file, offering superior mapping performance compared with other non-splicing mapping tools.

### 4.5. Gene Expression Calculation

The featureCounts (1.5.0-p3) software was employed to determine the number of reads aligned for each gene. The Fragments Per Kilobase of transcript per Million mapped reads (FPKM) value for each gene was subsequently calculated, incorporating both the gene length and the number of reads aligned to the gene. The FPKM value quantifies the expected number of fragments per kilobase of transcript sequence per million base pairs sequenced, accounting for both the sequencing depth and the influence of gene length on the read counts. This method remains one of the most commonly used techniques for estimating gene expression levels.

### 4.6. qRT-PCR Analysis

Total RNA was extracted from *P. persica* L. peel following the previously described protocol, and cDNA synthesis was performed from the total RNA in a reaction volume of 20 μL per the supplier’s instructions using a reverse transcription kit (Vazyme, CHINA). The coding sequence (CDS) of the relevant genes was obtained from the genome database (https://phytozome-next.jgi.doe.gov/) (accessed on 15 January 2024). Specific fluorescent quantitative primers were designed using Primer5.0, with each primer being approximately 20 bp in length and having a guanine–cytosine content ranging from 40% to 60%, melting temperature (Tm) values between 58–62 °C, and amplification product sizes spanning from 85 to 145 bp. The Primer-BLAST of NCBI (https://www.ncbi.nlm.nih.gov/ (accessed on 15 January 2024)) function was employed for the verification of primer specificity. The sequences of these primers are provided in Table S1. Actin (ppa007228mg) was chosen as the internal reference gene for *P. persica* L. (Brandi et al., 2011), and the data were analyzed using the 2^−ΔΔCT^ method [45] (the primers are depicted in Supplementary Table S1).

### 4.7. Identification and Sequence Analysis of PpPAO and PpSGR

The sequences of the *PpPAO* and *PpSGR* genes were retrieved from the Peach DataBase (http://www.peachmd.com/#/ (accessed on 15 January 2024)). Homologous protein sequences were identified and downloaded via BLASTp analysis using the NCBI database. Multiple sequence alignments of the *PpPAO* and *PpSGR* amino acid sequences from various species were performed using DNAMAN, and clustering analysis was conducted using Clustal W in MEGA7.0. Phylogenetic analysis, based on amino acid sequences, was executed using MEGA (version 5.02) with the neighbor-joining method, including 1000 bootstrap replicates [46].

### 4.8. Transient Transformation of N. benthamiana

The functional validation of *PpPAO* and *PpSGR* was performed via transient transformation of *N. benthamiana*. The plasmids for the transient expression analysis were constructed by inserting the full-length *PpPAO* (GenBank: ppa009783m) and *PpSGR* (GenBank: ppa010416m) genes into the pGreenII 62-sk vector. The pGreenII 62-sk vector was linearized through double digestion with BamHI and EcoRI restriction enzymes at 37 °C for 30 min in a water bath. Following the guidelines provided with the homologous recombination kit, recombination reactions were carried out between the purified *PpPAO* and *PpSGR* gene products and the linearized vectors to generate overexpression constructs for *PpPAO* and *PpSGR*. The resultant vectors (pGreenII 62-sk-PpPAO or pGreenII 62-sk-PpSGR) were then introduced into the *Agrobacterium tumefaciens* strain GV3101. The *N. benthamiana* plants were cultivated under standard greenhouse conditions at 22 °C with natural light, maintaining a 16 h light/8 h dark photoperiod. Plants with a minimum of six leaves were selected for infiltration and kept in the greenhouse throughout the experimental period. *Agrobacterium* cultures containing pGreenII 62-sk-PpPAO or pGreenII 62-sk-PpSGR were infiltrated into the leaves of *N. benthamiana* as described by Sainsbury et al. [47]. Control plants were infiltrated with the empty pGreenII 62-sk vector. Photographs of the leaves were taken three days post-infiltration (primers are listed in Supplementary Table S2).

### 4.9. Protein–Protein Interaction Experiments

The Y2H assay was conducted using the Matchmaker Gold Yeast Two-Hybrid System kit (Clontech, CA, USA) per the supplier’s guidelines. The coding sequences (CDSs) of *PpPAO* and *PpSGR* were inserted into the PDHB1 and PPR3-N vectors.

## 5. Conclusions

The appearance quality of *P. persica* L. serves a pivotal function in consumer selection and purchase decisions, with particular emphasis on the ground color of the fruit peel, which markedly influences initial consumer perceptions. The ground color is predominantly determined by the chlorophyll content. Degreening patterns in the peels of Zhongtao 14 and lines 20–29 were assessed, and KEGG enrichment analysis indicated the involvement of chlorophyll degradation genes within the purine metabolism pathway. qRT-PCR analysis revealed a notable upregulation in the expression of *PpPAO* and *PpSGR* from 8 to 0 days prior to maturation. Overexpression vectors for both *PpPAO* and *PpSGR* were developed, and transient transformation in *N. benthamiana* demonstrated that the overexpression of these genes induced considerable degreening in the leaves, with expression levels markedly higher than those in the control group. *PpPAO* and *PpSGR* were identified as potentially key regulators of the degreening process in the fruit peel of *P. persica* L. The Y2H assay further revealed an interaction between *PpPAO* and *PpSGR*.

## Figures and Tables

**Table 1:** The correlation coefficient between chlorophyll degradation genes and chlorophyll.

	CP14 Chlorophyll Content	20–29 Chlorophyll Content
*Pp* *NYC1*	0.193	−0.007
*Pp* *NOL*	0.005	−0.234
*Pp* *HCAR*	0.66	0.065
*Pp* *CLH1*	−0.541	−0.16
*Pp* *CLH2*	0.79 *	0.367
*Pp* *PPH*	0.612	−0.249
*Pp* *PAO*	−0.32	−0.776 *
*Pp* *RCCR*	−0.474	−0.591
*Pp* *SGR*	−0.522	−0.437
*Pp* *SGRL*	0.669	0.752
*Pp* *NYC1*	0.193	−0.007

Note: * indicate that the correlation reaches a significance level of 0.05.

## Data Availability

Data are available upon reasonable request to the corresponding author.

## Supplementary Materials

The following supporting information can be downloaded at: https://www.mdpi.com/article/10.3390/plants14030312/s1, Table S1: Primer sequences for qRT-PCR and gene cloning; Table S2. Primers used for gene-specific clone assay.

## Abbreviations

Chl: chlorophyll; CLH: chlorophyllase; DEG: differentially expressed gene; DGE: digital gene expression; GO: gene ontology; HCAR: hydroxy-Chl a reductase; KEGG: Kyoto Encyclopedia of Genes and Genomes; MAPK: mitogen-activated protein kinase; MCS: metal-chelating substance; NCCs: nonfluorescent Chl catabolites; NOL: NYC1-LIKE; NYC1: NON-YELLOW COLORING 1; PAO: pheide a oxygenase; pFCC: primary fluorescent Chl catabolite; Pheide: pheophorbide; PPH: pheophytinase; RCC: red Chl catabolite; RCCR: RCC reductase; SGR: stay-green protein.
