# Supplementary material for: Expression Analysis of Chlorophyll-Degradation-Related Genes in Prunus persica L. Peel and the Functional Verification of Key Genes
Source: Plants (Basel). 2025 Jan 21;14(3):312. doi: 10.3390/plants14030312 (PMC11821065; doi:10.3390/plants14030312)
Supplement: Supplementary file 1 [file plants-14-00312-s001.zip › plants-3394461-Supplementary.pdf]

Supplementary Table S1. Primer sequences for qRT-PCR

Gene	primer（5′–3′）	purpose
PpActin	GATTCCGGTGCCCAGAAGT	qRT-PCR
	CCAGCAGCTTCCATTCCAA	
PpNYC1	ATCGTGTGGTTGTCGCTTCT	qRT-PCR
	CAGGTGCTTAGAGGAGGCAC	
PpNOL	ATACGGGGCAACAAAGCGTA	qRT-PCR
	ACCATTCCTGGCGACAAGTT	
PpHCAR	CAGTGGAAATCGCCAACCAT	qRT-PCR
	AACTTTGGGGCAGGTTCAGG	
PpCLH1	CATGCCAAAACTGCCCTGTC	qRT-PCR
	AGGATATGGGGCCTGGTTCT	
PpCLH2	TCTCACGGCTTCATTGTCGT	qRT-PCR
	TGAACATGGGGTGGAAGCAA	
PpPPH	AGACTCGGGGCTTAGTAGCA	qRT-PCR
	CGCTCCGTCTCTGACAAACT	
PpPAO	AGGCAACCCACGGATTACTG	qRT-PCR
	AGTCTTCCCTGGTGCCATTG	
PpRCCR	ACATCCGCAGTGTTGTGTCT	qRT-PCR
	ATCCAGCCAAATTCCCAGCA	
PpSGR	GCTGTTGCTTCCCACCATTG	qRT-PCR
	TGTTTCTTGGGTTTGGCCCT	
PpSGRL	TGACGTGGTTGCAGAATGGA	qRT-PCR
	GCCAGGTCCAGCATGAGATT	
NtActin	GGCTTACATTGCTCTTGACTATGAAC	qRT-PCR
	ATCAGGCAGCTCGTAGCTCTTCT	

Supplementary Table S2. Primers used for gene-specific clone assay.

62-sk-*PpPAO*	atcgataagcttgatatcgaattcCAGCATGGAAGTACTCTCATACAGTTC	gene cloning
	ggccgctctagaactagtggatccTCATGATTTGAGCTCTAATTTCCTAAG	
62-sk-*PpSGR*	ggccgctctagaactagtggatccATGGGTACTTTGACTGCTGCTT	gene cloning
	atcgataagcttgatatcgaattcTTAGTTTGTTTCTTGGGTTTGGCCC	
